# tRNA Binding to Antitumor Drug Doxorubicin and Its Analogue

**DOI:** 10.1371/journal.pone.0069248

**Published:** 2013-07-29

**Authors:** Daniel Agudelo, Philippe Bourassa, Marc Beauregard, Gervais Bérubé, Heidar-Ali Tajmir-Riahi

**Affiliations:** Department of Chemistry and Physics, University of Québec at Trois-Rivières, Trois-Rivières, Québec, Canada; University of Hyderabad, India

## Abstract

The binding sites of antitumor drug doxorubicin (DOX) and its analogue *N*-(trifluoroacetyl) doxorubicin (FDOX) with tRNA were located, using FTIR, CD, fluorescence spectroscopic methods and molecular modeling. Different binding sites are involved in drug-tRNA adducts with DOX located in the vicinity of A-29, A-31, A-38, C-25, C-27, C-28, G-30 and U-41, while FDOX bindings involved A-23, A-44, C-25, C-27, G-24, G-42, G-53, G-45 and U-41 with similar free binding energy (-4.44 for DOX and -4.41 kcal/mol for FDOX adducts). Spectroscopic results showed that both hydrophilic and hydrophobic contacts are involved in drug-tRNA complexation and FDOX forms more stable complexes than DOX with *K*_DOX-tRNA_ = 4.7 (±0.5)×10^4^ M^−1^ and *K*_FDOX-tRNA_ = 6.3 (±0.7)×10^4^ M^−1^. The number of drug molecules bound per tRNA (*n*) was 0.6 for DOX and 0.4 for FDOX. No major alterations of tRNA structure were observed and tRNA remained in A-family conformation, while biopolymer aggregation and particle formation occurred at high drug concentrations.

## Introduction

**Figure 1:**
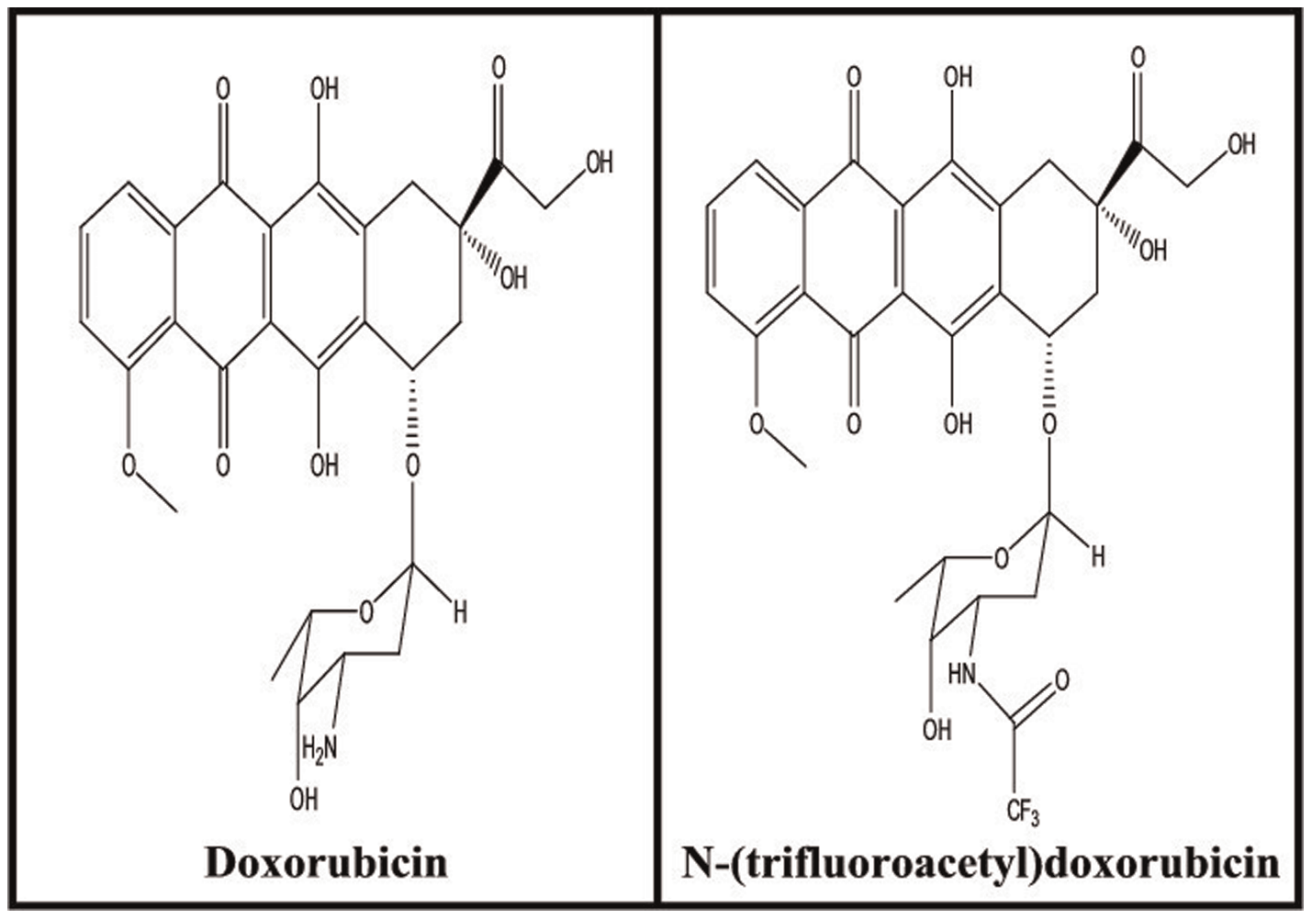
Chemical structures of doxorubicin (DOX) and *N*-(trifluoroacetyl) doxorubicin (FDOX).

Anthracycline antibiotics such as doxorubicin and its derivatives (Fig. 1) are extensively used as chemotherapeutic agents for the treatment of several types of cancers including leukemias, lymphomas, breast, uterine, ovarian, and lung cancers [1]. Doxorubicin intercalates into DNA duplex preventing DNA replication and transcription [2]. However, the use of doxorubicin has been limited by a dose-related and irreversible cardiotoxicity as well as by the emergence of drug resistance [3]. While doxorubicin intercalation with DNA duplex is well investigated [4], less is known about the effect of this antitumor drug on RNA structure. A comparative study of polynucleotides, DNA, RNA and nucleosomes with anthracycline antibiotics has been reported [5] and the effect of antibiotics on RNA synthesis and cell growth is investigated [6]–[8]. It has been shown that anthracyclines decrease RNA-binding activity and alter protein-RNA interactions [9.10]. Similarly, the double-stranded section of mRNA is the target of antibiotic binding, which alters iron responsive elements in mRNAs [11]. Recent report shows also the perturbation of microRNAs in rat heart during chronic doxorubicin treatment [12]. Even though much is known about the interaction of anthracyclines with DNA [2], the binding sites of antibiotics with RNA are not well characterized. Therefore, it was of interest to locate the binding sites of doxorubicin with tRNA and the effect of drug complexation on RNA structure and dynamic. In this study, we also verify the impact of a relatively subtle molecular modification on the daunosamine moiety of doxorubicin towards its interaction with tRNA. Thus, using drug analogue FDOX (Fig. 1) with blocked NH_2_ group helps us to understand the role of NH_2_ in drug efficacy and drug-RNA complexation. We recently investigated the interactions of DOX and FDOX with DNA [13]. It was of interest to further study these molecules and their interactions with tRNA. This comprehensive structural analysis of doxorubicin-tRNA adducts could have major biological and biochemical implications.

We report the structural analysis of tRNA complexes with DOX and FDOX by FTIR, CD, fluorescence spectroscopic methods and molecular modeling. Structural information regarding the doxorubicin binding to tRNA and the effect of drug on RNA stability and conformation are provided. To our knowledge, this is the first spectroscopic and structural analysis of tRNA interaction with DOX and FDOX at molecular level, which contributes to elucidating the effect of this class of antibiotics on RNA structure.

## Materials and Methods

### Materials

Doxorubicin hydrochloride was generously provided by Pharmacia/Farmitalia Carlos Erba, Italy and *N*-(trifluoroacetyl) doxorubicin was synthesized according to the published methods [14], [15]. Transfer RNA from Baker's yeast was purchased from Sigma Chemical Co., and used as supplied. The *A*260/*A*280 ratio of tRNA was 2.2, indicating that the tRNA was sufficiently free from protein [16].

### Preparation of stock solutions

Sodium-tRNA was dissolved to 1 % w/w (10 mg/ml) in Tris-HCl (pH 7.3) at 5°C for 24 h with occasional stirring to ensure the formation of a homogeneous solution. The final concentration of the stock tRNA solution was determined spectrophotometrically at 260 nm by using molar extinction coefficient of 6600 cm^−1^ M^−1^ (expressed as molarity of phosphate groups) [17], [18]. The UV absorbance at 260 nm of a diluted solution (40 μM) of tRNA used in our experiments was measured to be 0.25 (path length was 1 cm) and the final concentration of the stock tRNA solution was calculated to be 25 mM in tRNA phosphate. The solutions of DOX and FDOX (0.15 μM to 1 mM) were prepared in water for DOX and in ethanol/water 25/75% for FDOX and diluted in Tris-HCl (pH 7.4). The drug solution was added dropwise to tRNA solution with constant stirring to ensure the formation of homogeneous solution.

### FTIR spectroscopic measurements

Infrared spectra were recorded on a BOMEM DA3-0.02 Fourier transform infrared spectrometer, equipped with a nitrogen cooled HgCdTe detector and a KBr beam splitter. Solution spectra were recorded in solution on AgBr windows with resolution of 2 cm^−1^ and 100 scans with drug concentrations 0.125. 0.25 and 0.5 mM and a final tRNA concentration of 12.5 mM (P). The water subtraction was carried out with 0.1 M NaCl solution used as a reference at pH 7.3 [19]. A good water subtraction was achieved as shown by a flat baseline around 2200 cm−1 where the water combination mode is located. This method is a rough estimate, but removes the water content in a satisfactory way. The difference spectra [(tRNA solution + drug) – (tRNA solution)] were obtained, using the sharp tRNA band at 968 cm^−1^ as internal reference. This band, which is due to ribose C-C stretching vibrations, exhibits no spectral changes (shifting or intensity variation) upon drug-RNA complexation, and cancelled out upon spectral subtraction. The spectra are smoothed with Savitzky-Golay procedure [19].

### CD spectroscopy

Spectra of tRNA and its drug complexes were recorded at pH 7.4 with a Jasco J-720 spectropolarimeter. For measurements in the Far-UV region (200–320 nm), a quartz cell with a path length of 0.01 cm was used. Three scans were accumulated at a scan speed of 50 nm per minute, with data being collected at every nm from 200 to 320 nm. Sample temperature was maintained at 25°C using a Neslab RTE-111 circulating water bath connected to the water-jacketed quartz cuvettes. Spectra were corrected for buffer signal and conversion to the Mol CD (Δε) was performed with the Jasco Standard Analysis software. The drug concentrations used in our experiment varied from 125 μM to 500 μM with the final tRNA concentration of 2.5 mM.

### Fluorescence spectroscopy

Fluorimetric experiments were carried out on a Perkin-Elmer LS55 Spectrometer. Stock solution of drug (20 μM) in Tris-HCl (pH 7.4) was also prepared at 24±1°C. Various solutions of tRNA (1 to 200 μM) were prepared from the above stock solutions by successive dilutions at 24±1°C. Samples containing 0.06 ml of the above drug solution and various tRNA solutions were mixed to obtain final tRNA concentrations ranging from 1 to 100 μM with constant drug content (20 μM). The fluorescence spectra were recorded at λ_ex_  =  480 nm and λ_em_ from 500 to 750 nm. The intensity of the band at 592 nm from doxorubicin and its analogue [20] was used to calculate the binding constant (K) as reported [21]–[26].

On the assumption that there are (*n*) substantive binding sites for quencher (*Q*) on protein (*B*), the quenching reaction can be shown as follows:
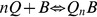
(1)


The binding constant (*K_A_*), can be calculated as:
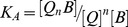
(2)where, [*Q*] and [*B*] are the quencher and polymer concentration, respectively, [*Q_n_B*] is the concentration of non fluorescent fluorophore-quencher complex and [B_0_] gives total polymer concentration:



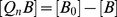
(3)

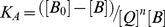
(4)


The fluorescence intensity is proportional to the polymer concentration as described:
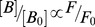
(5)


Results from fluorescence measurements can be used to estimate the binding constant of drug-polymer complex. From eq 4:
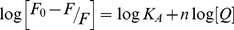
(6)


The accessible fluorophore fraction (*f*) can be calculated by modified Stern-Volmer equation: 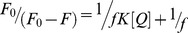
(7)where, *F*_0_ is the initial fluorescence intensity and *F* is the fluorescence intensities in the presence of quenching agent (or interacting molecule). *K* is the Stern-Volmer quenching constant, [Q] is the molar concentration of quencher and *f* is the fraction of accessible fluorophore to a polar quencher, which indicates the fractional fluorescence contribution of the total emission for an interaction with a hydrophobic quencher [27], [28]. The *K* will be calculated from 
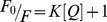
.

### Molecular modeling

The docking studies were performed with ArgusLab 4.0.1 software (Mark A. Thompson, Planaria Software LLC, Seattle, Wa, http://www.arguslab.com). tRNA structure were obtained from the PDB (ID: 6TNA) [29] and the three dimensional structures of DOX and FDOX were generated from PM3 semi-empirical calculations using Chem3D Ultra 6.0. The docking runs were performed on the ArgusDock docking engine using high precision with a maximum of 150 candidate poses. The conformations were ranked using the Ascore scoring function, which estimates the free binding energy. Upon docking of drug to RNA, the current configurations were optimized using a steepest decent algorithm until convergence, within 40 iterations and nucleobase residues within a distance of 3.5 Å relative to drug were involved in the complexation.

## Results

### FTIR spectra of drug-tRNA complexes

**Figure 2:**
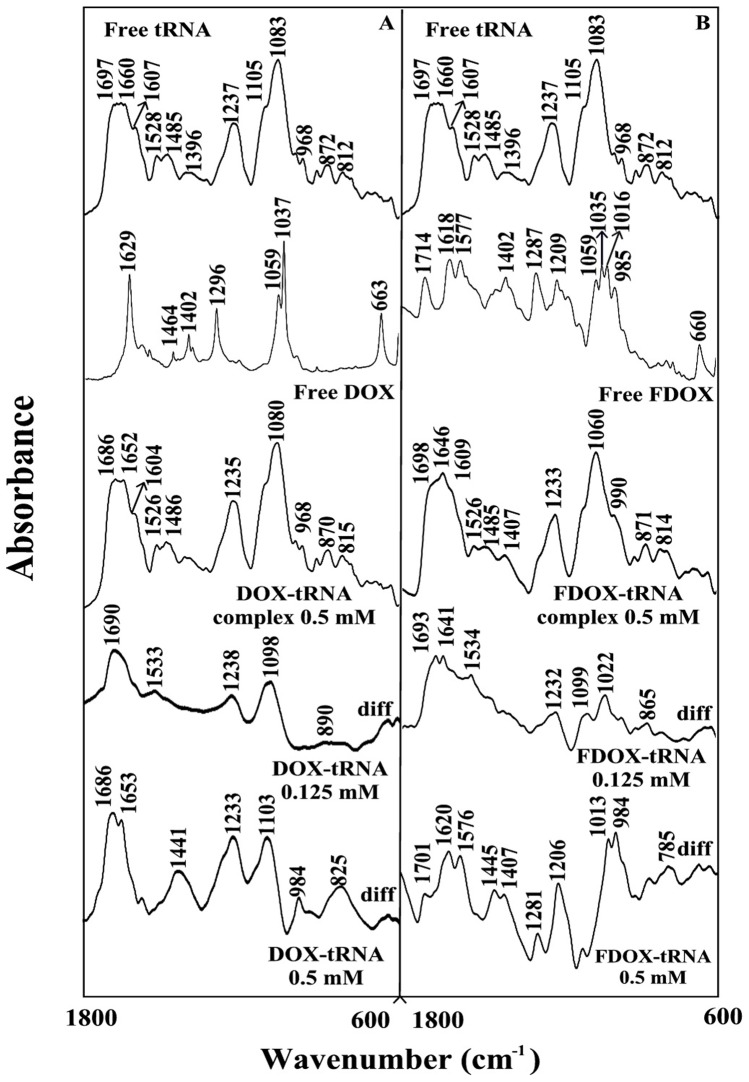
FTIR spectra and difference spectra [ (tRNA solution + drug solution) – (tRNA solution)] in the region of 1800-600 cm^−1^ for the free tRNA and its DOX (A) and FDOX (B) complexes in aqueous solution at pH 7.4 with various drug concentrations and constant tRNA content (12.5 mM).

The infrared spectral changes observed for drug-tRNA interaction are presented in Figure 2. complexation of DOX and FDOX with tRNA brought major spectral changes for tRNA in-plane vibrational frequencies [30]–[34]. Spectral shifting and intensity increase were observed for the guanine band at 1697, uracil band at 1660, adenine band at 1607 and cytosine vibrations at 1528 and 1485 cm^−1^, upon drug complexation (Fig. 2). The guanine band at 1697 shifted at 1686 (DOX) and 1698 cm^−1^ (FDOX), while uracil band at 1660 appeared at 1652 (DOX) and 1646 cm^−1^ (FDOX) upon drug interaction (Fig. 2, complexes 0.5 mM). The adenine band at 1607 was observed at 1604 (DOX) and 1609 cm^−1^ (FDOX), while cytosine band at 1528 and 1485 cm^−1^ showed no major shifting on drug-tRNA complexation (Fig. 2, 0.5 mM complexes). The shifting was accompanied by a major intensity increase for these vibrations as shown by positive spectral features at 1701-1686 (guanine) and 1653-1620 (uracil), in the difference spectra of DOX and FDOX-tRNA adducts (Fig. 2, diffs, 0.125 and 0.5 mM). Drug interaction also induced spectral changes for tRNA phosphate group as the band at 1237 (PO_2_ asymmetric) and 1083 cm^−1^ (PO_2_ symmetric) gained intensity and shifted to a lower frequency at 1080-1060 cm^−1^, in the spectra of drug-tRNA complexes (Fig. 2, complexes 0.5 mM). These spectral changes are due to drug interactions with RNA nucleobases (guanine, uracil and adenine) and the backbone phosphate group. Similar infrared spectral changes were observed for tRNA vibrational frequencies upon biogenic polyamine complexation [30].

### CD spectra and tRNA conformation

**Figure 3:**
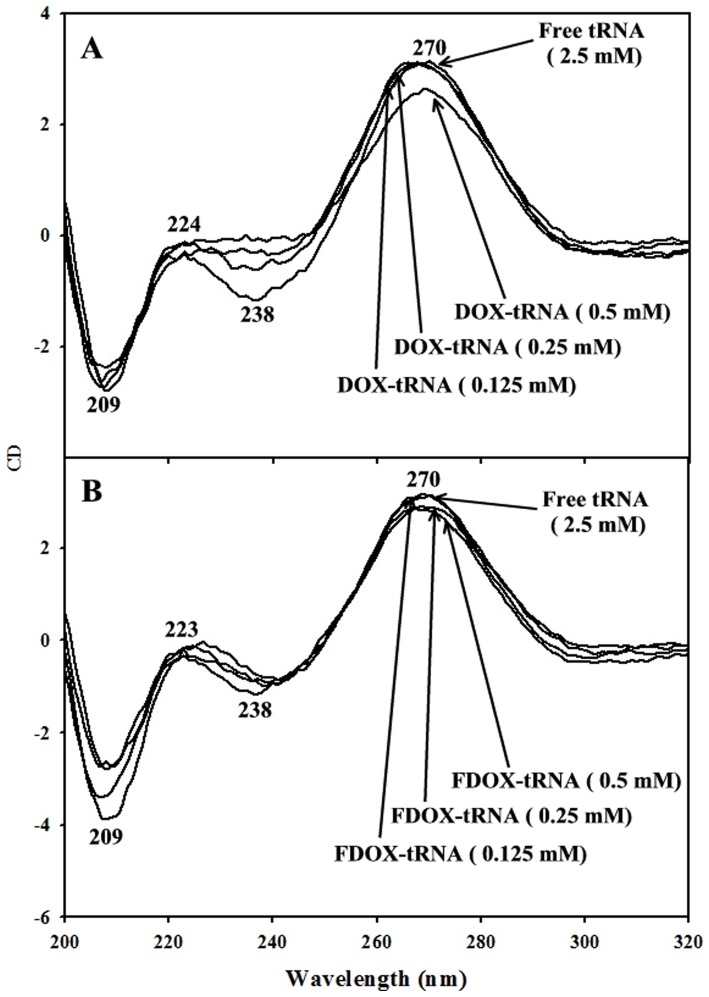
Circular dichroism of the free tRNA and its DOX (A) and FDOX complexes (B) in aqueous solution with 2.5 mM tRNA concentration and 0.125 to 0.5 mM drug concentrations at pH 7.4.

The CD spectra of the free tRNA with DOX and FDOX complexes at different drug concentrations are shown in Figure 3. The CD spectrum of the free tRNA composed of four major peaks at 209 (negative), 224 (positive), 238 (negative) and 270 nm (positive) (Fig. 3). This is consistent with CD spectra of double tRNA in A- conformation [35], [36]. Upon DOX and FDOX complexation (Fig. 3, 0.125 and 0.25 mM), no major alterations of CD bands were observed, while a decrease in intensity of CD bands was observed at high drug concentration (Fig. 3, 0.5 mM). The lack of spectral shifting indicates of tRNA remains in A conformation, while the reduction in the intensity of CD bands is related to tRNA aggregation and particle formation. Similar CD spectral changes were observed for tRNA aggregation and particle formation in the presence of cationic lipids, dendrimers and PEG nanoparticles [37]–[39].

### Fluorescence spectra and stability of drug-tRNA complexes

**Figure 4:**
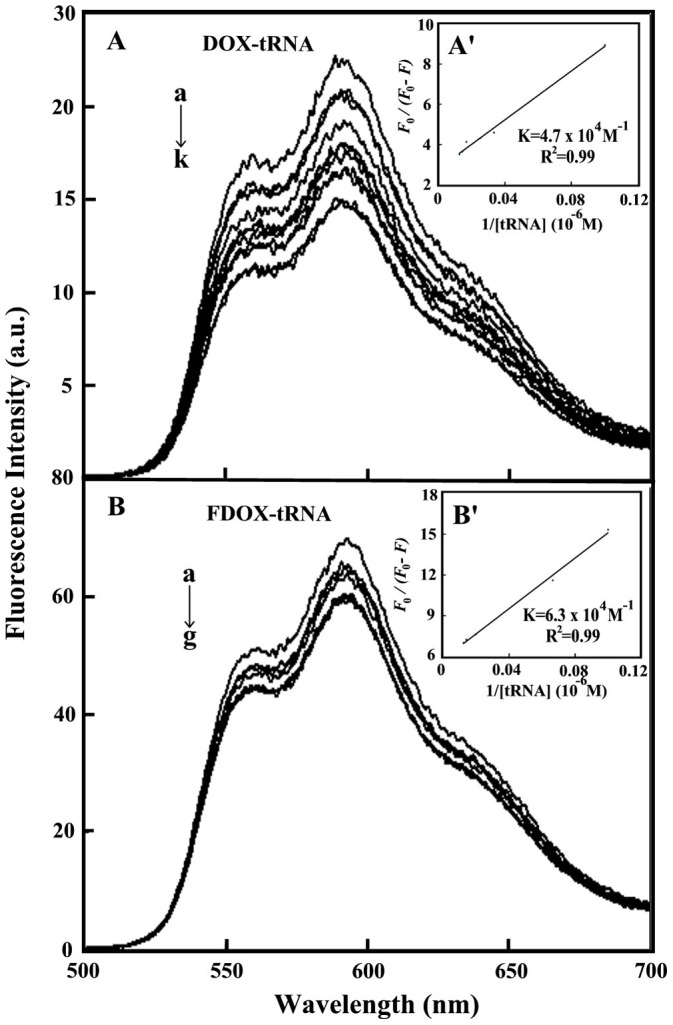
Fluorescence emission spectra of drug-tRNA systems in 10 mM Tris-HCl buffer pH 7.4 at 25°C for A) DOX-tRNA: (a) free DOX (20 μM), (b-k) with DOX-tRNA complexes at 5, 10, 15, 20, 30, 40, 50, 60, 70 and 80 μM; B) FDOX-tRNA: (a) free FDOX (20 μM), (b-gj) with FDOX-tRNA complexes at 5, 10, 20, 30, 40 and 60 μM. Inset: *F*_0_ / (*F*_0_ – *F*) vs 1 / [tRNA] for A' (DOX-tRNA) and B' (FDOX-tRNA).

Since tRNA is a weak fluorophore, the titrations of DOX and FDOX were done against various tRNA concentrations, using drug excitation at 480 nm and emission at 500–750 nm [20]. When drug interacts with tRNA, fluorescence may change depending on the impact of such interaction on the drug conformation, or *via* direct quenching effect. The decrease of fluorescence intensity of DOX or FDOX has been monitored at 592 nm for drug-tRNA systems (Fig. 4A and 4B). The plot of 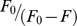 vs 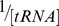 is shown in Fig. 4A'and 4B'. Assuming that the observed changes in fluorescence come from the interaction between the drug and tRNA, the quenching constant can be taken as the binding constant of the complex formation. The *K* value obtained is the averages of four and six-replicate run for drug-polymer systems. Each run involves several different concentrations of tRNA (Fig. 4A and 4B). The overall binding constants were *K*_DOX-tRNA_ = 4.7 (±0.5)×10^4^ M^−1^ and *K*_FDOX-tRNA_ = 6.3 (±0.7)×10^4^ M^−1^ (Fig. 4A' and 4B') showing more stable complexes formed with FDOX than DOX.

The *f* value calculated from Eq. 7 represents the mole fraction of the accessible population of fluorophore to quencher. The *f* values were from 0.20 to 0.50 for these drug-tRNA complexes indicating a large portion of fluorophore was exposed to quencher.

**Figure 5:**
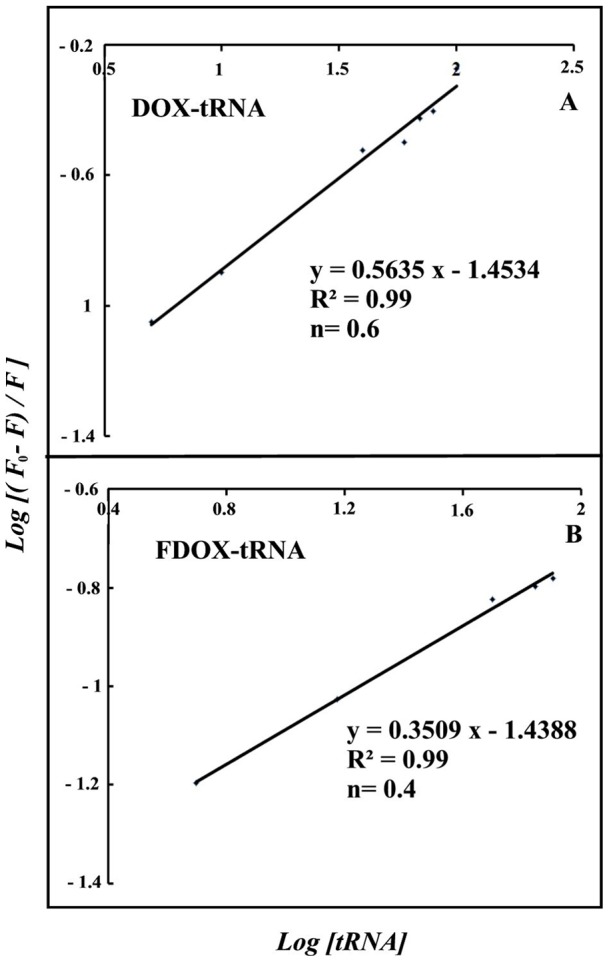
The plot of 
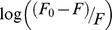
as a function of 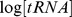 for calculation of number of bound drug molecule (*n*) per tRNA (A) DOX-tRNA and (B) FDOX-tRNA complexes.

The number of drug molecules bound per tRNA (*n*) is calculated from 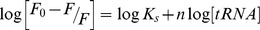 for the static quenching [40]–[43]. The *n* values from the slope of the straight line plot showed 0.6 (DOX) and 0.4 (FDOX) drug molecules that are bound per tRNA molecule (Fig. 5).

**Figure 6:**
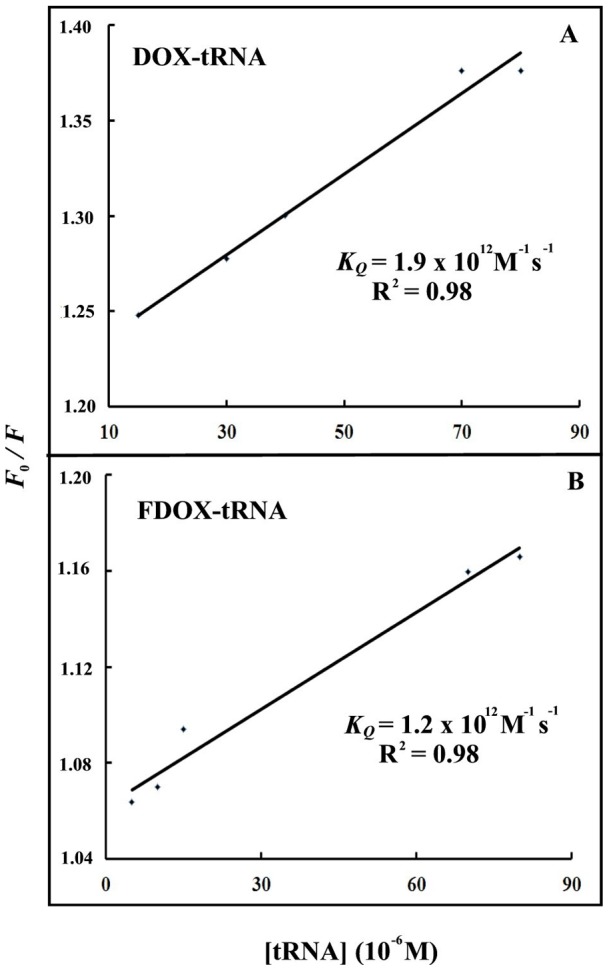
Stern-Volmer plots of fluorescence quenching constant (*K_Q_*) for the tRNA and its drug complexes at different tRNA concentrations (A) DOX-tRNA and (B) FDOX-tRNA complexes.

In order to verify the presence of static or dynamic quenching in drug-tRNA complexes we have plotted *F_0_/F* against *Q* to estimate the quenching constant (*KQ*) and the results are show in Figure 6. The plot of *F_0_/F* versus Q is a straight line for drug*-*tRNA adducts indicating that the quenching is mainly static in these drug-biopolymer complexes (Fig. 6). The quenching constant *K_Q_* was estimated according to the Stern-Volmer equation: 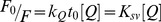
(8)where *F_0_* and *F* are the fluorescence intensities in the absence and presence of quencher, [Q] is the quencher concentration and *K*_sv_ is the Stern-Volmer quenching constant [34], [35], which can be written as 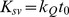; where *k_Q_* is the drug quenching rate constant and t_0_ is the lifetime of the fluorophore in the absence of quencher about 1.1 ns for free DOX and FDOX around neutral pH [20]. The quenching constants (*K_Q_*) are 1.9×10^12^ M^−1^/s for DOX*-*tRNA and 1.2×10^12^M^−1^/s for FDOX-tRNA complexes (Fig. 6). Since these values are greater than the maximum collisional quenching constant (2.0×10^10^ M^−1^/s), the static quenching is dominant in these drug-tRNA complexes [44]–[46].

### Docking study

**Figure 7:**
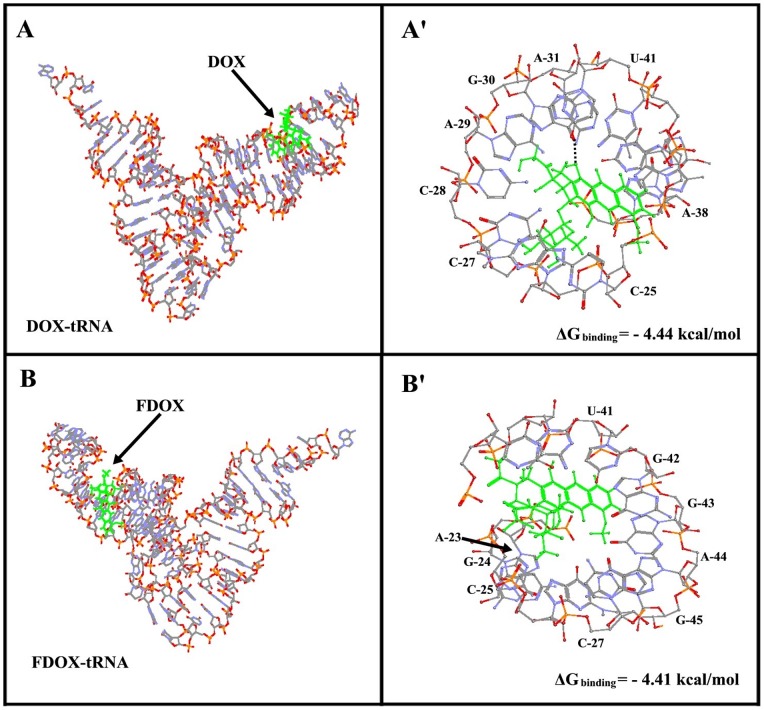
Best conformations for drug docked to tRNA (PDB entry 6TNA). The drug is shown in green color. (A) shows tRNA in stick model with DOX in sticks and (A') shows DOX binding sites represented in sticks with the corresponding base residues (B) shows tRNA in stick model with FDOX in sticks and (B') FDOX binding sites represented in sticks.

Our spectroscopic results are accompanied by docking study in which the DOX and FDOX were docked to tRNA to determine the preferred binding sites on tRNA. The dockings results are shown in Figure 7 and Table 1. The models show that both DOX and FDOX do not share similar binding sites on tRNA with DOX surrounded by A-29, A-31, A-38, C-25, C-27, C-28, *G-30 (H-bonding with doxorubicin NH_2_ group) and U-41 with the free binding energy of -4.44 kcal/mol (Fig. 7 and Table 1). FDOX is in the vicinity of A-23, A-44, C-25, C-27, G-24, G-42, G-43, G-45 and U-41 with the free binding energy of -4.41 kcal/mol (Fig. 7 and Table 1). The binding energy (ΔG) shows more stable complexes formed with DOX than FDOX (Table 1). The extra stability is related to the presence of H-bonding between guanine-30 and the NH_2_ group in DOX-tRNA complexes (not present in FDOX-tRNA adduct). This further confirms the importance of the free daunosamine amino group in DOX for its optimal interaction with DNA and induction of its biological activity [13]. The results presented in the latter study were shown to parallel the biological activities of DOX and FDOX on SXC01 human tumour cancer cells [47].

**Table 1:** Ribonucleotides in the vicinity of DOX and FDOX docked with tRNA (PDB 6TNA) and the free binding energies of the docked complexes.

Complex	Ribonucleotides involved in drug interactions	ΔG _binding_ (Kcal/mol)
DOX-tRNA	A-29, A-31, A-38, C-25, C-27C-28, *G-30, U-41.	−4.44
FDOX-tRNA	A-23, A-44, C-25, C-27, G-24G-42, G-43, G-45, U-41.	−4.41

* Hydrogen bonding involved with this nucleotide.

## Discussion

Doxorubicin remains as one of the most effective chemotherapeutic anticancer drugs of the past 50 years and is crucial to the treatment of a range of neoplasms including acute leukemia, malignant lymphoma, and breast cancer [1]–[3]. However, like all the other anticancer drugs, the efficacy of DOX is associated with high systemic toxicity to healthy tissue [48]. While doxorubicin intercalation with DNA duplex is well investigated [4], less is known about the effect of this antitumor drug on RNA structure. A comparative study of polynucleotides, DNA, RNA and nucleosomes with anthracycline antibiotics has been reported [5], [6] and the effect of antibiotics on RNA synthesis and cell growth is investigated [7], [8].

Even though the intercalation of doxorubicin into DNA duplex has been well investigated [4], [49], little is known about the important role of free NH_2_ group in drug-DNA and drug-tRNA interactions. Furthermore, most of the anthracyclines marketed in the world today doxorubicin, daunorubicin, idarubicin, epirubicin, pirarubicin and zorubicin contain a free amino functional group, which is very important for drug-DNA interactions [50]. Thus, a part of our study was aimed to determine the importance of the free daunosamine amino group in DOX-tRNA interactions and its biological implication. Therefore, the amino group of daunosamine was acetylated with S-ethyltrifluoroacetate under standard reaction conditions [13]. This particular derivative was selected in order to change the physico-chemical property of the amino group (likely to from H-bonds) into an amide that reduces drastically the basic character of the nitrogen atom. Our infrared results showed no major differences between DOX and FDOX interactions with tRNA since both are groove binders (Fig. 2). However, our CD spectroscopic results showed more aggregation of tRNA by DOX than FOX, while tRNA remained in A-conformation (Fig. 3) while, drug binding to DNA induced a partial B to A-DNA conformational transition [13]. Furthermore, molecular modeling showed different binding sites for DOX and FDOX on tRNA with the presence of H-bonding between guanine-30 and drug NH_2_ group in DOX-tRNA, which is not present in FDOX-tRNA adduct (Table 1). Such H-bonding network brought more stability for DOX-tRNA complex than FDOX-tRNA adduct (Table 1). It is concluded that these structural changes are responsible for antitumor activity of doxorubicin and are consistent with the antitumor activities of DOX and FDOX obtained on SXC01 human tumour cancer cells [47].

In conclusion, DOX and FDOX bind tRNA *via* both hydrophilic and hydrophobic contacts. Drug binding induces biopolymer aggregation and particle formation, while tRNA remains in A-conformation. The presence of NH_2_ group on DOX causes only minor differences between DOX and FDOX interactions with tRNA. Our results reveals major differences between drug-DNA binding (intercalation) [13] and drug-tRNA interaction (non-intercalation) with DOX that is yet again forming more stable complexes than FDOX.
